# Fine Root Growth of Black Spruce Trees and Understory Plants in a Permafrost Forest Along a North-Facing Slope in Interior Alaska

**DOI:** 10.3389/fpls.2021.769710

**Published:** 2021-11-16

**Authors:** Kyotaro Noguchi, Yojiro Matsuura, Tomoaki Morishita, Jumpei Toriyama, Yongwon Kim

**Affiliations:** ^1^Tohoku Research Center, Forestry and Forest Products Research Institute (FFPRI), Morioka, Japan; ^2^Research Planning Department, Forestry and Forest Products Research Institute (FFPRI), Tsukuba, Japan; ^3^Kyushu Research Center, Forestry and Forest Products Research Institute (FFPRI), Kumamoto, Japan; ^4^International Arctic Research Center, University of Alaska Fairbanks, Fairbanks, AK, United States

**Keywords:** active layer thickness, feather moss, fine root morphology, ingrowth core, *Sphagnum* moss

## Abstract

Permafrost forests play an important role in the global carbon budget due to the huge amounts of carbon stored below ground in these ecosystems. Although fine roots are considered to be a major pathway of belowground carbon flux, separate contributions of overstory trees and understory shrubs to fine root dynamics in these forests have not been specifically characterized in relation to permafrost conditions, such as active layer thickness. In this study, we investigated fine root growth and morphology of trees and understory shrubs using ingrowth cores with two types of moss substrates (feather- and *Sphagnum* mosses) in permafrost black spruce (*Picea mariana*) stands along a north-facing slope in Interior Alaska, where active layer thickness varied substantially. Aboveground biomass, litterfall production rate, and fine root mass were also examined. Results showed that aboveground biomass, fine root mass, and fine root growth of black spruce trees tended to decrease downslope, whereas those of understory Ericaceae shrubs increased. Belowground allocation (e.g., ratio of fine root growth/leaf litter production) increased downslope in both of black spruce and understory plants. These results suggested that, at a lower slope, belowground resource availability was lower than at upper slope, but higher light availability under open canopy seemed to benefit the growth of the understory shrubs. On the other hand, understory shrubs were more responsive to the moss substrates than black spruce, in which *Sphagnum* moss substrates increased fine root growth of the shrubs as compared with feather moss substrates, whereas the effect was unclear for black spruce. This is probably due to higher moisture contents in *Sphagnum* moss substrates, which benefited the growth of small diameter (high specific root length) fine roots of understory shrubs. Hence, the contribution of understory shrubs to fine root growth was greater at lower slope than at upper slope, or in *Sphagnum* than in feather-moss substrates in our study site. Taken together, our data show that fine roots of Ericaceae shrubs are a key component in belowground carbon flux at permafrost black spruce forests with shallow active layer and/or with *Sphagnum* dominated forest floor.

## Introduction

Permafrost ecosystems store huge amounts of carbon below ground (about 1,000 Pg), which accounts for 50% of global belowground organic carbon pool (Tamocai et al., 2009; Mishra et al., 2021). Although frozen soil can be a harsh environment for vascular plants, coniferous forests dominated with black spruce (*Picea mariana*) (Interior Alaska, Northwestern Canada) and larch (*Larix gmelinii* and *L. cajanderi*) (central and eastern Siberia) are widely distributed in permafrost regions (Viereck et al., 1983; Abaimov, 2010; Fujii et al., 2020), covering more than 20% of the boreal forest globally (Osawa and Zyryanova, 2010). Considering this large area of distribution, it is important to improve our knowledge of carbon dynamics in permafrost forests to better understand terrestrial carbon cycling. However, the information on plant growth in permafrost forests is still limited especially for belowground parts, although a few papers reported above and belowground productivity in those permafrost forests (Kajimoto et al., 1999; Ruess et al., 2003).

One of the dominant species of permafrost forests is black spruce (*Picea mariana*), which is well known to distribute widely across boreal zone of North America (Vogel et al., 2008). This wide distribution can be explained by high adaptability of this species to wide ranges of environmental conditions. One of the unique characteristics of black spruce stands in permafrost regions appears to be high carbon partitioning below ground. For example, it was reported that root-to-shoot biomass ratio (R/S ratio) in a permafrost black spruce stand was about 0.88 (Noguchi et al., 2012), which is three times as large as averaged R/S ratio of boreal forests (about 0.27) (Cairns et al., 1997). Furthermore, fine root production rates in other black spruce stands on permafrost were estimated to be 126–374 g m^–2^ year^–1^, which were 14–17 times as large as litterfall production rates in those stands (Ruess et al., 2003). These data suggest that root growth dynamics are a key to understanding how the black spruce can survive in harsh environments such as cold and poorly drained permafrost (Viereck et al., 1983). However, it should be noted that most of the data on fine root production reported previously have been stand-level assessments that include both black spruce and understory plants (Ruess et al., 1996, 2003; O’Connell et al., 2003; Bond-Lamberty et al., 2004; Kalyn and Van Rees, 2006). Since understory plants were reported to make a significant contribution to aboveground productivity in permafrost or poorly drained black spruce stands (O’Connell et al., 2003; Ruess et al., 2003; Bond-Lamberty et al., 2004), it would be valuable to evaluate fine root growth of understory plants separately from black spruce trees to better understand how the belowground carbon flux is controlled in permafrost forests.

Permafrost conditions vary with latitudinal location or topography. Throughout Interior Alaska, permafrost is found on north-facing slopes and poorly drained lowlands (Viereck et al., 1983), where the thickness of the active layer (mineral soil layer with seasonal thaw) varies with slope position and aspect (Noguchi et al., 2016; Tanaka-Oda et al., 2016; Wolken et al., 2016). For example, it was reported that the active layer thickness and biomass of black spruce trees are greater in the upper slope than in lower slope, whereas biomass allocation to fine roots (i.e., fine root/aboveground biomass ratio) was greater in the lower slope (Noguchi et al., 2016). These results likely reflect acclimation of black spruce trees to colder and less fertile soils in the lower slope position with thinner active layer thickness (Tanaka-Oda et al., 2016).

Another factor influencing growth of trees on permafrost would be mosses and lichens which typically cover forest floor continuously and play an important role in regulating biophysical conditions of thick organic layer (O’Donnell et al., 2009; Terrier et al., 2014; Pacé et al., 2020), where more than 90% of black spruce fine roots were observed in the above-mentioned permafrost forest (Noguchi et al., 2016). Species composition of the forest floor moss community varies with environmental conditions such as drainage and canopy openness. Particularly, *Sphagnum* mosses dominate poorly drained wet sites whereas feather mosses, such as *Pleurozium schreberi* and *Hylocomium splendens*, dominate sites with better drainage (Bisbee et al., 2001). A recent paper reported that the mosses affected the growth of black spruce seedlings differently by species, in which 2-year-old seedlings grew better on feather mosses than on *Sphagnum* mosses, although such difference was not observed in 3-year-old seedlings planted in the field (Pacé et al., 2018).

Understory shrubs are known to often contribute significantly to aboveground productivity in black spruce forests (Ruess et al., 2003). However, little is known about the separate contributions of overstory trees and understory shrubs to fine root dynamics. Although a recent study showed greater contribution of understory plants to fine root biomass at a shallow than at a deep active layer stand (Noguchi et al., 2016), information is limited on how fine root growth of the trees and the shrubs vary in relation to gradients in abiotic and biotic conditions such as slope position, active layer thickness, and species composition of forest floor mosses. Thus, the first objective of this study was to quantify fine root growth rates of black spruce and understory plants in mature black spruce stands with varied active layer thickness across slope positions. Based on the previous reports on soil fertility (Tanaka-Oda et al., 2016) and fine root biomass (Noguchi et al., 2016) in our study site, we hypothesized that (1) fine root growth rate was lower but belowground allocation was greater on lower slopes with colder and less fertile soils and (2) contribution of understory plants to total fine root growth was greater on lower slopes. The second objective was to elucidate effects of different moss substrates on the fine root growth rates. Considering the data from seedling experiments (Pacé et al., 2018), we also hypothesized that (3) fine root growth was greater in feather than in *Sphagnum*-moss substrates. Finally, our third objective was to quantify variation in fine root morphology such as specific root length (SRL) across slope positions or in different moss substrates, which could explain how black spruce trees and understory shrubs acclimated to the varied abiotic and biotic conditions on permafrost.

## Materials and Methods

### Study Site

This study was conducted in a ca. 100-year-old black spruce (*Picea mariana*) forest on a north facing slope in the Caribou Poker Creek Research Watershed (CPCRW) of University of Alaska Fairbanks (65°08′N, 147°30′W). The mean annual temperature and precipitation are −2.5°C and 400 mm, respectively (Petrone et al., 2006). This is the same black spruce forest studied by Noguchi et al. (2016) and Tanaka-Oda et al. (2016). We established one 14 m × 14 m plot at each of the upper, middle, and lower slope positions, where the elevation is 360, 330, and 260 m, respectively (Supplementary Figures 1, 2). Hereafter, these plots will be designated as NE360, NE330 and NE260, respectively. The stand characteristics and photographs of the study plots are shown in Table 1 and Supplementary Figure 3, respectively. Variations of environmental conditions across the three plots included 8–14 degrees in slope inclination, 34–42 cm in organic layer thickness, 63–113 cm in active layer thickness, and 3.1–4.6 g kg^–1^ in total soil nitrogen in A horizon of mineral soil. Stand densities and mean diameter at breast height (DBH) of black spruce trees taller than 1.3 m varied as 5,420–9,220 trees ha^–1^ and 2.7–5.1 cm, respectively. Major species of understory vascular plants included Ericaceae shrubs such as *Rhododendron* and *Vaccinium* spp. (Noguchi et al., 2016). Forest floor was continuously covered by feather mosses (*Pleurozium schreberi* and *Hylocomium splendens*), *Sphagnum* mosses and lichens.

**TABLE 1:** Stand characteristics of the black spruce stands in this study.

**Plot**		**NE360**	**NE330**	**NE260**
Slope aspect		NE	NE	NE
Slope position		Up slope	Mid slope	Low slope
Elevation	(m)	360	330	260
Slope inclination	(degree)	8	8	14
Organic layer thickness	(cm)	34 ± 3	42 ± 2	42 ± 2
Active layer thickness^*^	(cm)	113 ± 10	63 ± 5	74 ± 7
Soil N concentration^*^	(g kg**^–^**^1^)	4.6	3.6	3.1
Organic layer temperature^**^				
Under feather moss	(°C)	8.8	8.9	7.9
Under *Sphagnum* moss	(°C)	No data	9.4	7.0
Black spruce (H > 1.3 m)				
Stand density	(trees ha**^–^**^1^)	8,660	9,220	5,420
Mean DBH	(cm)	5.1 ± 0.2 x	2.9 ± 0.1 y	2.7 ± 0.2 y
Mean Height^*^	(m)	7.1 ± 0.6	4.5 ± 0.2	3.0 ± 0.2
Black spruce (H 0.5–1.3 m)				
Stand density	(trees ha**^–^**^1^)	2,210	9,150	8,700
Mean D_0_	(cm)	1.2 ± 0.1	1.7 ± 0.1	1.9 ± 0.1
Aboveground biomass				
Black spruce	(kg m**^–^**^2^)	5.96	2.08	1.11
Understory plants	(g m**^–^**^2^)	43.6 ± 10.8 a	145 ± 31 b	142 ± 18 b
Forest floor cover^***^				
Feather moss	(%)	89	81	41
*Sphagnum* moss	(%)	2	16	38
Other	(%)	9	3	21

*Data from adjacent stands (Tanaka-Oda et al., 2016).

**Mean growing season temperature (May–September 2018–2019) in the depth of 10 cm.

***Data of NE360 and NE330 were from Noguchi et al. (2016). Different alphabetical letters indicate significant difference between the plots (a,b: Tukey-Kramer HSD test; x,y: Steel-Dwass test).

### Vegetation Survey

To estimate the aboveground biomass of black spruce trees, DBH was measured for all trees taller than 1.3 m in the three plots in August 2018. As for trees 0.5–1.3 m in height, diameter at stem base was measured in eight 2 m × 2 m subplots in each 14 m × 14 m plot. Aboveground biomass was then calculated using allometric equations established in Noguchi et al. (2012). To estimate the aboveground biomass of understory vascular plants, 50 cm × 50 cm plots were set at six locations around the side of each 14 m × 14 m plot and aboveground parts of the plants were harvested in July 2018. Samples were dried at 75°C for 48 h and weighed. Forest floor cover was recorded at nine 2 m × 2 m subplots in each plot by visual inspection. Mean area proportions covered by feathermoss (*P. schreberi* and *H. splendens*), *Sphagnum* moss, and others (including lichens) were then calculated.

### Litterfall

Litterfall was collected using litter traps in the three plots. In September 2016, eight litter traps (20 cm × 27 cm basket) were set on the ground in each plot (Supplementary Figure 2). The litterfall samples were collected in September 2017, August 2018, and September 2019, transported to the laboratory, and divided into leaves, branches, and reproductive organs (e.g., cones) of black spruce trees, leaves of understory plants, and others. These samples were dried at 70°C for more than 48 h and weighed. Litterfall production rates of the three sampling periods were calculated and their averaged values were considered as the annual litterfall production rates. Data from one of the eight traps in NE360 were excluded from further analyses because it contained large amounts of debris of spruce shoots and cones dropped by squirrels, which was 13 times as large as the mean value from seven other traps (ca. 2002 vs. 157 g m^–2^ year^–1^).

### Fine Root Mass

To estimate fine root mass, core sampling was conducted in August 2018. In each plot, nine 2 m × 2 m subplots were selected, and one sample was taken from each subplot (Supplementary Figure 2). The core samples were collected by vertically inserting a metal tube (3.0 cm in inner diameter, 60 cm in length) to the depth of gravel or frozen soil that could not be penetrated. Maximum depth sampled by this procedure was 50 cm. The core samples were divided into organic and mineral soil layers and transported on ice to the laboratory and stored in a freezer until processed. From these core samples, organic layer thickness was calculated by subtracting the length of mineral soil core from the depth of tube insertion. In the laboratory, the core samples were spread in water and fine roots were manually picked up using tweezers. The samples were rinsed with water on a sieve with 0.5 mm openings to remove fine particles of soil and organic matter when necessary. The fine root samples were divided into those of black spruce trees and understory plants by their morphological characteristics (Noguchi et al., 2016). Those fine root samples were further divided into two groups by diameter classes of < 0.5 mm and 0.5–2.0 mm. Although we removed fragile roots as dead roots, a certain fraction of black or dark brown roots in the remaining samples might also have been dead. Therefore, we analyzed those fine root samples as “fine root mass” and not as “fine root biomass” in this study. Fine root samples were dried at 70°C for more than 48 h and weighed. Fine roots in mineral soil were not used for further analyses because more than 90 and 80% of fine roots of black spruce and understory plants, respectively, were present in the organic layer (data not shown).

### Fine Root Growth Rate

Fine root growth rate was examined for 2 years (September 2017 to September 2019) with an ingrowth core method. Although data obtained by the ingrowth core method have been previously expressed as “fine root production rate” (Vogt et al., 1998), here, we described our data as “fine root growth rate” due to lack of information on fine root mortality and decomposition, which might contribute significantly to the amounts of fine root production (Hendricks et al., 2006; Osawa and Aizawa, 2012). Plastic mesh tubes with 2-mm openings, 3.2 cm in diameter, and 30 cm in length were used as the ingrowth cores. Two different substrates, dead feather mosses and dead *Sphagnum* mosses, were used. For the substrate preparation, moss shoots were collected near the study plots and packed gently in the mesh tubes after removing their top living (green) parts. Care was taken to remove any live or dead roots from the moss substrates prior to packing. Ingrowth cores were installed on 26–27 September 2017 at nine subplots in each of the NE360, NE330, and NE260 plots (Supplementary Figure 2). To prepare the installation holes, a metal tube smaller than the ingrowth cores (2.4 cm in diameter) was used to ensure good contact between the ingrowth core and surrounding soft organic layer. At NE330 and NE260 plots, a pair of ingrowth cores with feather and *Sphagnum*-moss substrates were set 20–55 cm apart in each subplot. At NE360, only feather moss was used to pack the ingrowth cores because the ground cover by *Sphagnum* mosses was little at the NE360 plot and surrounding area (Table 1).

After 2 years of incubation, the ingrowth cores were collected on September 19–22, 2019. During sampling, the ingrowth cores were removed together with surrounding organic layer by cutting with hedge shears followed by removal of organic matter and roots outside the ingrowth cores using scissors. The ingrowth cores were divided into three 10 cm depth intervals and transported to the laboratory on ice and stored in a freezer until processed. Fine roots in the ingrowth cores were collected using the same procedure for above-mentioned fine root mass analyses. Roots that appeared to be fresh were separated as living roots based on their color and tensile strength for four ingrowth core samples from NE360 (feather moss substrate) and for four pairs of the samples from NE330 and NE260 (a pair included the feather and *Sphagnum*-moss substrates at a given sampling location). These living roots were subjected to analyses of fine root morphology as described below. After the root separation and morphological analyses, the samples were dried at 70°C for 48 h and weighed. Fine root growth rate was calculated as fine root mass divided by area of the ingrowth cores (8.0 cm^2^) and by the incubation period (2 years). Since fine root growth was examined with feather and *Sphagnum* moss substrates, weighted mean of fine root growth rate was also calculated for the NE330 and NE260 plots considering proportion of ground cover by feather (F) and *Sphagnum* (Sp) mosses (except for “Other”). The F:Sp ratios used for the calculations were 0.84:0.16 and 0.52:0.48 (Table 1) in the NE330 and NE260, respectively.

### Fine Root Morphology

Living roots collected from the ingrowth cores were spread in water on a glass petri dish. Then, samples were scanned using a flat-bed scanner (GT-980X, Epson, Suwa, Japan) at 800 dpi under constant brightness (80 in Epson Scan software) and back-lighting using a LED light panel (Treviewer A3-500, Trytec, Kyoto, Japan). The obtained root images were analyzed for length, volume, and diameter using the WinRHIZO Pro software (Regent Instruments, Quebec, Canada). From these data, specific root length (SRL, m g^–1^) was calculated as the fine root length (m sample^–1^) divided by fine root dry weight (g sample^–1^), and root tissue density (RTD, g cm^–3^) was calculated as the fine root dry weight (g sample^–1^) divided by fine root volume (cm^3^ sample^–1^).

### Soil Temperature and Moisture Monitoring

Temperature and water content in the organic layer were monitored at 10 cm depth using sensors and data loggers (5TE and Em50, METER Group Inc., Pullman, WA, United States). In the NE360 plot, one sensor was set under a feather moss patch, while in the NE330 and NE260 plots, one sensor was installed under each of a feather and a *Sphagnum* moss patches. The data were retrieved from data loggers in August 2018 and September 2019.

### Statistics

One-way ANOVA was performed to examine variations of fine root parameters for each moss substrate, litterfall, and aboveground biomass of understory vascular plants across study plots (NE360, NE330 and NE260). When the variations were significant, Tukey–Kramer’s HSD test was performed for multiple mean comparisons. To examine the effects of the moss substrates (feathermoss vs. *Sphagnum* moss) on the fine root parameters, split-plot ANOVA was conducted for each of the NE330 and NE260 plots, in which sampling locations within a plot were considered as random effect. Before the ANOVA, normality and equal variances of the dataset were checked by Shapiro–Wilk test and Bartlett test, respectively. When the normality or equal variance were not confirmed, the data were subjected to Box-Cox transformation. However, if the transformed data did not meet these conditions, non-parametric Steel–Dwass test was performed. All the statistical analyses were done using JMP 12.0 (SAS Institute, Cary, NC, United States).

## Results

### Organic Layer Conditions

Organic layer thickness at NE360 plot (34 cm) was approximately 80% of that at the NE330 and NE260 plots (42 cm) (Table 1). Although temperature and volumetric water contents (VWC) were monitored by only one sensor per each ground cover type (feather or *Sphagnum*-mosses) in each plot, mean organic layer temperature during the growing seasons (May–September of 2018–2019, 10 cm depth) appeared lower at NE260 (7.0–7.9°C) than at NE360 and NE330 (8.8–9.4°C) (Table 1 and Supplementary Figure 4), while VWC were greater under *Sphagnum* mosses than under feather mosses (Supplementary Figure 4). The mean temperature (May–September of 2018) measured at bottom of the organic layer at adjacent stands of NE360, NE330, and NE260 were 7.4, 7.9, and 4.7°C, respectively (Supplementary Figure 5).

### Aboveground Biomass and Litterfall

Diameter at breast height (DBH) of black spruce trees was significantly greater at NE360 than at NE330 and NE260, where abovegound biomass of black spruce trees was 5.96, 2.08, and 1.11 kg m^–2^, respectively (Table 1). In contrast, aboveground biomass of understory vascular plants was significantly smaller at NE360 (ca. 44 g m^–2^) than at NE330 and NE260 (ca. 140 g m^–2^) (Table 1).

**FIGURE 1:**
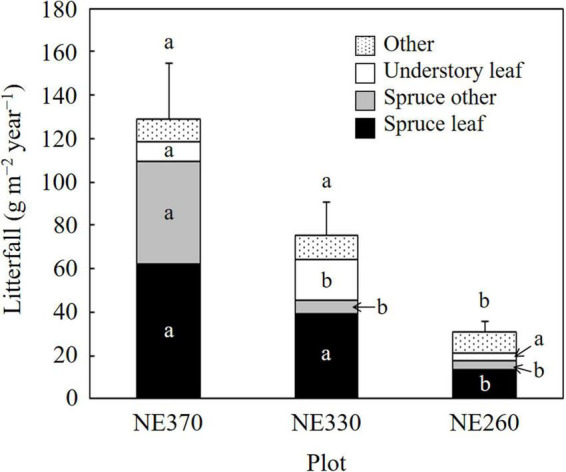
Litterfall production rates in NE360, NE330, and NE260 plots. Black, gray, white, and dotted columns represent data on leaves of black spruce, other organs of black spruce, leaves of understory plants and others, respectively. Data shown are mean + SE (*N* = 7–8). Different alphabetical letters indicate significant difference between the plots within each organ (Tukey–Kramer HSD test).

Total amounts of annual litterfall production ranged ca. 35–175 g m^–2^ year^–1^, which was significantly smaller at NE260 than at NE330 and NE360 (Figure 1). Leaf litters of black spruce trees and understory plants accounted for 35–48 and 5–23%, respectively, of the litterfall. In general, litterfall from black spruce trees was greater in the order of NE360 > NE330 > NE260, whereas leaf litter production of understory plants was significantly greater at NE330 than in two other plots (Figure 1).

### Fine Root Mass

**FIGURE 2:**
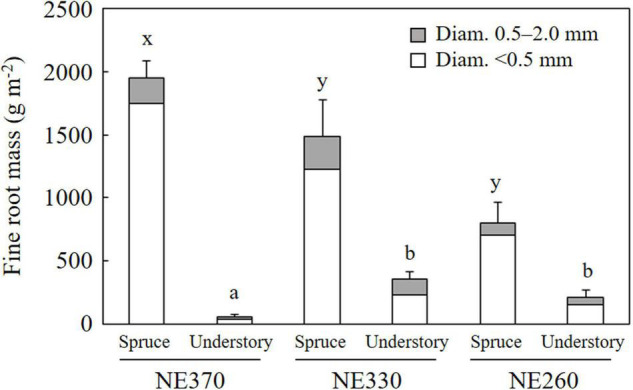
Fine root mass of black spruce and understory plants in NE360, NE330, and NE260 plots. White and gray columns represent data on roots < 0.5 and 0.5–2.0 mm in diameter. Data shown are mean + SE (*N* = 9). Different alphabetical letters indicate significant difference between the plots for total fine root mass in < 2 mm in diameter (a–b, Tukey–Kramer HSD test; x–y, Steel–Dwass test).

At NE360, NE330 and NE260 plots, mean fine root mass (Diam. < 2.0 mm) of black spruce trees was 1,949, 1,489, and 804 g m^–2^, respectively, while that of understory plants was 54, 352, and 212 g m^–2^, respectively (Figure 2). The fine root mass at NE360 was significantly larger than in the NE330 and NE260 for black spruce trees, whereas that of understory plants was significantly smaller than at NE330 and NE260. Roots < 0.5 mm in diameter accounted for 81–90% of the fine root mass of black spruce trees, while that of understory plants accounted for 67–85%.

### Fine Root Growth Rate

**FIGURE 3:**
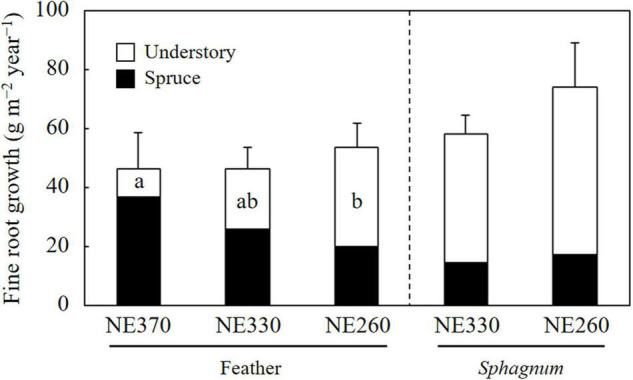
Fine root growth rates of black spruce and understory plants in feather or *Sphagnum*-moss substrates at NE360, NE330, and NE260 plots. Black and white columns represent data on black spruce and understory plants, respectively. Data shown are mean + SE (*N* = 9). Different alphabetical letters represent significant difference between the plots within each species category in each moss substrate (Tukey–Kramer HSD test). No letter was given when significant variation was not detected among the plots by one-way ANOVA.

**FIGURE 4:**
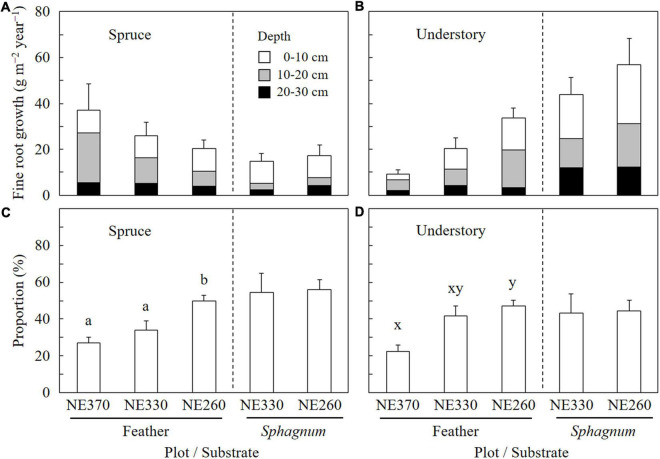
Vertical distribution of fine root growth of black spruce and understory plants in feather or *Sphagnum*-moss substrates at NE360, NE330, and NE260 plots [**(A)** fine root growth rates of black spruce; **(B)** those of understory plants; **(C)** proportions of fine root growth at the depth of 0–10 cm in total fine root growth (0–30 cm) of black spruce; **(D)** those of understory plants]. White, gray, and black columns represent data in the depths of 0–10, 10–20, and 20–30 cm, respectively **(A,B)**. Data shown are mean + SE (*N* = 9). Different alphabetical letters indicate significant difference between the plots within each moss substrate (a–b, Tukey–Kramer HSD test; x–y, Steel–Dwass test). Data on fine root growth rates **(A,B)** were not subjected to the statistical analyses.

In the feather moss substrate, fine root growth rate of black spruce trees tended to decrease downslope, which ranged from 20.3 to 37.0 g m^–2^ year^–1^ (Figure 3). In contrast, the fine root growth rate of understory plants increased downslope significantly, which ranged from 9.3 to 33.7 g m^–2^ year^–1^. As a result, variation of total fine root growth rate among the plots was little, ranging from 46.3 to 54.0 g m^–2^ year^–1^. In the *Sphagnum* moss substrate (NE330 and NE260), fine root growth rates of black spruce, understory plants, and total were 14.7–17.2, 43.8–57.0, and 58.5–74.2 g m^–2^ year^–1^, respectively, which were not significantly different between the plots (Figure 3). As for the fine root growth in feather moss substrate, the proportion of fine root growth in the depth of 0–10 cm to total fine root growth rate (0–30 cm) was significantly greater at NE260 than at NE360 for both black spruce trees (27 vs. 50%) and understory plants (23 vs. 47%) (Figure 4). In contrast, for the *Sphagnum* moss substrate, the proportions were similar between the two plots (NE330 and NE260), averaging 54%–56% and 43%–44% in black spruce and understory plants, respectively.

When fine root growth rate was compared between the different moss substrates, it was significantly greater in *Sphagnum* moss than in feather moss for understory plants at NE330 (Table 2). Although the difference was not significant, a similar trend was observed at NE260 (split plot ANOVA, *p* = 0.094). The fine root growth rate of black spruce and total fine root growth rate were not significantly different between the moss substrates.

**TABLE 2:** Results of split-plot ANOVA for effects of different moss substrates on fine root parameters.

		**NE330**		**NE260**	
		***F*-value**	***P*-value**	***F*-value**	***P*-value**
FRG	Spruce	5.22	0.052	0.24	0.64
	Understory (U)	**23.7**	**0.001**	3.61	0.094
	Total (T)	2.94	0.12	1.79	0.22
	U/T	**37.5**	** < 0.001**	4.74	0.061
SRL	Spruce	0.00	0.98	0.19	0.69
	Understory	0.08	0.79	0.34	0.60
RTD	Spruce	7.04	0.077	**35.2**	**0.010**
	Understory	0.08	0.80	2.38	0.22
Diameter	Spruce	0.08	0.79	0.09	0.79
	Understory	0.14	0.74	0.11	0.76

*U/T, proportion of fine root growth rate of understory plants in total fine root growth rate. Bold values indicate that the effect was significant (*p* < 0.05).*

We examined belowground allocation by calculating ratios of fine root growth rates to leaf litter production rates. Weighted means of fine root growth rates based on feather and *Sphagnum* moss ground cover were used as plot means at NE330 and NE260. The obtained ratios of black spruce, understory plants and total vascular plants were 0.59–1.41, 1.08–12.38, and 0.65–3.77, respectively, which were greater in the order of NE260 > NE330 > NE360 (Table 3).

**TABLE 3:** Weighted means of fine root growth rates (FRG), mean leaf litter production rates (LLP), and ratio of FRG/LLP in each plot.

	**Plot**	**Spruce**	**Understory**	**Total**
Fine root growth (FRG)	NE360	37.0	9.3	46.3
g m^–2^ year^–1^	NE330^*^	24.2	24.2	48.4
	NE260^*^	18.8	44.9	63.7
Leaf litter production (LLP)	NE360	62.4	8.6	71.0
g m^–2^ year^–1^	NE330	39.0	18.5	57.5
	NE260	13.3	3.6	16.9
FRG/LLP	NE360	0.59	1.08	0.65
	NE330	0.62	1.31	0.84
	NE260	1.41	12.38	3.77

*Fine root growth rate was calibrated by ground cover ratio between feather and *Sphagnum*-mosses for each of the NE330 and NE260 plot.

### Fine Root Morphology

**FIGURE 5:**
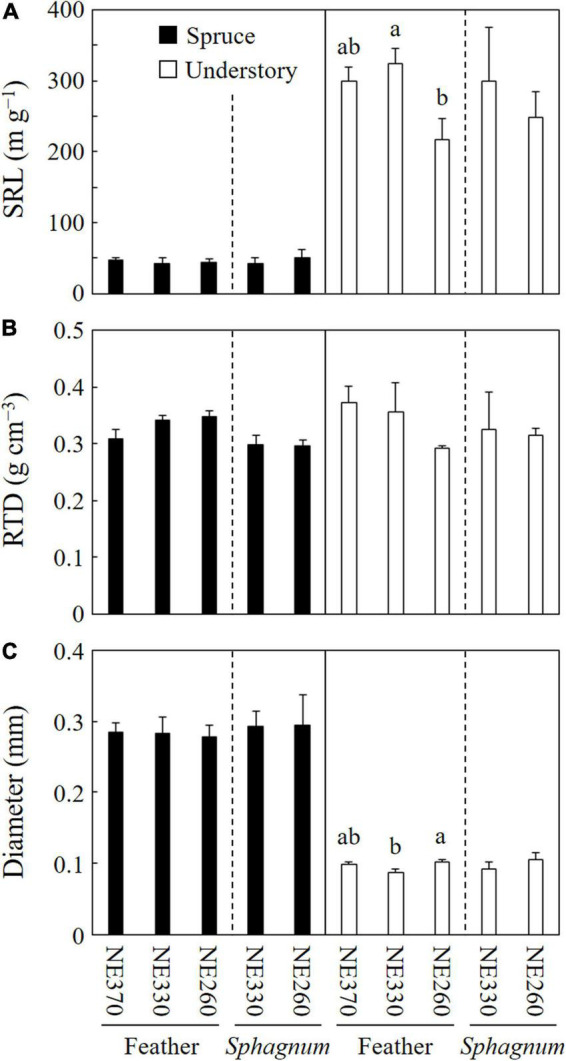
Fine root morphology of black spruce and understory plants in feather or *Sphagnum*-moss substrates at NE360, NE330, and NE260 plots. Specific root length [SRL, **(A)**], root tissue density [RTD, **(B)**], and root diameter **(C)** are shown. Black and white columns represent data on black spruce and understory plants, respectively. Data shown are mean + SE (*N* = 4). Different alphabetical letters indicate significant difference between the plots within each species category with each moss substrate (Tukey–Kramer HSD test). No letter was given when significant variation was not detected among the plots by one-way ANOVA.

Mean specific root length (SRL), root tissue density (RTD), and diameter of black spruce fine roots in the ingrowth cores were ca. 42–50 m g^–1^, 0.30–0.35 g cm^–3^, and 0.28–0.29 mm, respectively, while those of understory plants were ca. 217–325 m g^–1^, 0.29–0.37 g cm^–3^, and.09–0.11 mm, respectively (Figure 5). In feather moss substrates, the SRL and diameter of understory fine roots were significantly different among the plots, whereas those of black spruce trees were not. RTD in the feather moss was not significantly different among the three plots for either black spruce or understory plants. As for roots in *Sphagnum* moss, fine root morphology did not vary significantly between the plots (NE330 and NE260). At NE260, on the other hand, RTD of black spruce was significantly lower in *Sphagnum* moss than in feather moss (Table 2). Although the difference was not significant, a similar trend of variation was found in the NE330 (split plot ANOVA, *p* = 0.077).

### Contribution of Understory Plants

**FIGURE 6:**
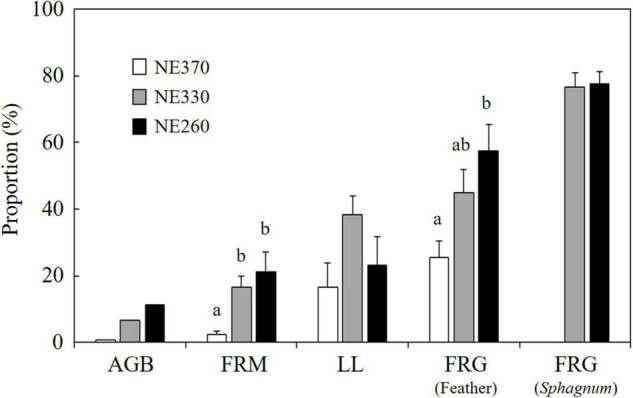
Proportion of understory plants in total (black spruce + understory) aboveground biomass (AGB), fine root mass (FRM), leaf litter production (LLP), and fine root growth (FRG) in feather and *Sphagnum*-moss substrates. White, gray, and black columns represent data from NE360, NE330, and NE260 plots, respectively. Data shown are mean + SE (AGB, *N* = 1; LLP, *N* = 7–8; FRM and FRG, *N* = 9). Different alphabetical letters indicate significant difference between the plots within each of the above and belowground parameters (Tukey–Kramer HSD test).

The contribution of understory plants to total stand aboveground biomass of vascular plants was relatively small across plots (0.7–11%) (Figure 6). However, the fine root mass of understory plants constituted between 2.1 and 18.1% of total stand fine root mass and was significantly lower at NE360 than at the other two plots. Stand-level contributions of understory plants to leaf litter and fine root growth rate averaged between 17–38% and 25–78%, respectively, in which the former was not significantly different across the plots, whereas the latter was significantly smaller at NE360 than at NE260. In addition, the contribution of understory plants to total fine root growth was significantly greater in *Sphagnum* substrate than in feather moss substrate at NE330 (Table 2). Although the difference was not significant, a similar trend was found at NE260 (Split plot ANOVA, *p* = 0.061).

## Discussion

### Fine Root Growth in Relation to the Slope Positions

In this study, we investigated fine root growth rates of black spruce and understory plants separately, across different slope positions, while most of the previous studies have reported total (black spruce + understory plants) fine root production rates (O’Connell et al., 2003; Ruess et al., 2003; Kalyn and Van Rees, 2006). Since the effects of different permafrost conditions were not well known, we focused on the variation of fine root growth across slope positions, where active layer thickness varied. Our results on aboveground biomass, fine root mass, litterfall production rates, and fine root growth rates show that above and belowground growth of black spruce trees were smaller at lower slope than at upper slope (Figures 1–3 and Table 1). However, the variation of fine root growth rates of black spruce was less evident (not significant) as compared with those of aboveground parameters. As a result, ratios of fine root growth rates to leaf litter production rates, for example, were greater at a lower slope, especially at NE260 plot (Table 3). These results suggest that belowground allocation of black spruce trees increased downslope, which supports our hypothesis 1. Similarly, our results show that the belowground allocation of understory plants was also greater at the lower slope than at upper slope (Table 3). However, in contrast to those of black spruce trees, above and belowground (bio) mass and growth of understory plants were significantly greater at the lower slope (NE330 and/or NE260 plots) than at NE360 (Table 1 and Figures 1–3). For example, the fine root growth rate of understory plants in feather moss at NE260 (33.7 g m^–2^ year^–1^) was 3.6 times as large as that at NE360 (9.3 g m^–2^ year^–1^). Thus, as for understory plants, our results supported the hypothesis 1 for belowground partitioning, although the amount of fine root growth was greater at the lower slope, in contrast to our expectation.

Similar patterns of change in belowground allocation have often been observed in studies on other non-permafrost forests. For example, fine root biomass, growth, or allocation (e.g., fine root biomass per basal area) were negatively correlated with nitrogen mineralization rates (temperate deciduous broad-leaved forest) (Tateno et al., 2004) and annual precipitation (European beech forests) (Hertel et al., 2013) or positively correlated with soil C/N ratio (boreal-temperate Norway spruce, Scots pine and silver birch forests) (Ostonen et al., 2017). In our study site, Tanaka-Oda et al. (2016) showed that soil nitrogen concentration tended to be lower in the lower than in upper-slope. Furthermore, they demonstrated that foliar δ^15^N of black spruce trees positively correlated with aboveground biomass and current shoot growth rates, suggesting that contribution of ectomycorrhizal fungi in nitrogen uptake increased when aboveground growth of black spruce trees was limited. Thus, greater belowground allocation at lower slope was likely driven in part by a response to lower nutrient availability (Table 3).

As mentioned above, fine root growth of understory plants was significantly greater at the lower slope than at the upper slope, although that of black spruce trees showed an opposite trend of variation along the slope. As a result, the contribution of understory plants to total fine root growth increased downslope, which supported our hypothesis 2. One of the reasons for the difference between the black spruce and understory plants in the patterns of variation in fine root growth was probably in a gradient of light availability for understory plants. Since lower stand density with smaller black spruce trees formed an open canopy stand (Table 1 and Supplementary Figure 3), light availability for understory plants would be greater at the lower slope than at the upper slope, which could translate to increased plant growth. Another explanation could be differences in responses to belowground conditions. Although the proportion of fine root growth in surface organic layer (0–10 cm depth) was greater at the lower slope for both black spruce and understory plants, the former resulted from a decrease at deeper layer (10–30 cm depth), whereas the latter was due to increase in the fine root growth at surface layer (0–10 cm depth) (Figure 4). This suggests a possibility that the negative effects of belowground conditions (e.g., low temperature, Supplementary Figures 4, 5) were little or negligible for understory plants as compared with those for black spruce trees. Thus, it seemed that variations of fine root growth across slope positions were controlled by mixed effects of above and belowground resource availability, although further study is needed to clarify the underlying mechanisms including competition between the plant species (Inderjit and Mallik, 1996).

### Effects of Forest Floor Mosses on Fine Root Growth

As discussed above, smaller aboveground biomass of black spruce trees at lower slope could be linked to lower belowground temperature due to higher permafrost table in our study site (Table 1 and Supplementary Figures 4, 5). The results on the vertical distribution of fine root growth of black spruce trees showed that surface concentration of the fine root growth was more evident at lower slope than at upper slope, which might result from growth suppression of fine roots at the deeper layers with lower temperature (Figure 4 and Supplementary Figure 5). However, our data on stand characteristics suggested that both active layer and organic layer thickness were similar between the low-slope (NE260) and mid-slope (NE330) plots, even though aboveground biomass of black spruce trees in the former plot was about 50% of that in the latter (Table 1). Therefore, other factors should also be considered to explain the growth variation of black spruce and understory plants across the slope positions.

In this study, we focused on differences in ground cover, which was highly heterogeneous along the toposequence, but in general, the proportion of *Sphagnum* moss relative to feather moss increased downslope (Table 1; Morishita et al., 2019; Toriyama et al., 2021). Our measurements investigating effects of different moss substrates showed that while fine root growth of black spruce was not strongly sensitive to the different moss substrates, *Sphagnum* moss positively affected fine root growth of understory plants as compared with feather moss (Table 2). Thus, our hypothesis 3 was not supported by the results of this study. A recent study using black spruce seedlings suggested that *Sphagnum* moss substrate could affect the growth of black spruce negatively due to lower nutrient availability, although the effects were likely to vary depending on growth conditions (greenhouse vs. field) and/or seedling characteristics (age) (Pacé et al., 2018). In our study, fine root growth of black spruce tended to be lower in *Sphagnum* than in feather-mosses at the NE330 (*p* = 0.052), but not at the NE260 (Table 2). There is a possibility that lower nutrient availability or lower pH within the *Sphagnum* moss substrates may decrease fine root growth of black spruce, but further study is needed to know whether the *Sphagnum* moss substrates have negative effects on the growth of black spruce fine roots. Another well-known characteristic of *Sphagnum* moss substrates is that they can retain higher amounts of water than feather moss substrates (O’Donnell et al., 2009; Terrier et al., 2014). This moisture condition could benefit fine root growth of understory plants more than that of black spruce, considering that fine roots of understory plants such as Ericaceae shrubs were much thinner and probably more vulnerable to occasional drought in feather moss substrate than those of black spruce (Figure 4 and Supplementary Figure 4).

As for fine root morphology, root tissue density (RTD) of black spruce fine roots was lower in the *Sphagnum* than in feather-moss substrates, although we did not examine it at NE360 in this study (Table 2). This suggested that fine roots of black spruce in *Sphagnum* moss substrates might have higher physiological activity than in feather moss substrates (Makita et al., 2015). In contrast, such an effect was not observed in understory fine roots. Previous studies showed that RTD increased with decreasing soil resource availability (Makita et al., 2015; Kramer-Walter et al., 2016). Although nutrient availability in feather moss substrates is likely higher than in *Sphagnum* moss substrates (Pacé et al., 2018), occasional drought in feather moss might result in higher RTD in feather than in *Sphagnum* moss substrates in our study (Table 2 and Supplementary Figure 4). However, a recent study reported that drought effect on RTD was not so simple, in which drought treatment increased RTD of distal (1st and 2nd order) roots of *Quercus alba*, while opposite responses were observed in higher (4th) order roots, and the effects varied with tree species (Suseela et al., 2020). Hence, further study is needed to understand why RTD of black spruce varied with the different moss substrates in our study site.

Taken together, our results suggested that black spruce responded to different moss substrates by changing fine root morphology (e.g., RTD) rather than the fine root growth rate in our study site, whereas the effects on understory plants were more evident in fine root growth rate than in fine root morphology (Figure 5 and Table 2).

### Implication to Carbon Dynamics

One of previous papers using the minirhizotron technique showed that fine root production rates in three permafrost black spruce stands in Interior Alaska averaged 126–374 g m^–2^ year^–1^, which were 14–17 times as large as litterfall production rates (9–22 g m^–2^ year^–1^) (Ruess et al., 2003). This suggests that the contribution of fine roots to net primary productivity (NPP) or belowground carbon input is much greater than leaves or aboveground litters, respectively. On the other hand, our results showed that fine root growth rates were 46–64 g m^–2^ year^–1^ (Table 3), which were 0.26–1.8 times as large as aboveground litterfall production rates (35–176 g m^–2^ year^–1^; Figure 1). These results suggested that the contribution of fine roots to NPP or belowground carbon input in permafrost black spruce stands varied with stand productivity, in which the contribution of fine roots was greater at lower productivity sites (Table 3; Ruess et al., 2003). Here, it should be noted that methods might also affect the estimates of fine root growth (production) rates. In general, fine root production rates estimated by the minirhizotron technique were greater than those by the ingrowth core method (Finér et al., 2011), which were also demonstrated in mature black spruce stands in Canada (Steele et al., 1997). This is probably because the ingrowth core method cannot detect mortality and decomposition of fine roots (turnover), which are, of course, occurring throughout the ingrowth period (Hendricks et al., 2006; Osawa and Aizawa, 2012).

We found that the contribution of understory plants to total stand-level fine root mass and fine root growth rates were greater at lower slope than at upper slope (Figures 2, 3). Since SRL of understory plants was six times that of black spruce trees (Figure 5), an increased proportion of understory fine roots would likely enhance carbon flux through fine root turnover and respirations, because roots with smaller diameters generally have a shorter life span and greater physiological activity (Wells and Eissenstat, 2001; Pregitzer et al., 2002; Ruess et al., 2006; Makita et al., 2015). In our results, ratios of the fine root mass to the fine root growth rate of understory plants across the plots (4.7–15) were smaller than those of black spruce trees (43–53) (Figure 2 and Table 3), suggesting that fine root turnover of understory plants was faster than that of black spruce trees. These findings suggest that belowground carbon flux in permafrost black spruce forests may increase by increasing the productivity of understory plants (under improved light conditions) with faster fine root turnover at shallower permafrost sites. Some previous studies on boreal forests estimated fine root (litter) production rates of trees and understory plants separately, in which, for example, understory plants accounted for 19–21% (black spruce forests; Steele et al., 1997), 6–29% (drained fen or bog forests with Scots pine, etc.; Bhuiyan et al., 2017), and 20–47% (Scots pine forests; Ding et al., 2021) of total fine root (litter) production, which were comparable or smaller than our data on understory contribution to fine root growth (25–78%; Figure 6). Therefore, to better understand belowground carbon flux in permafrost forests, it is recommended that future studies will focus more on the roles of understory plants such as Ericaceae shrubs.

Here, it should be noted that our data were obtained only from one experiment for 2 years. Although long-term data on fine root growth is not available, recent studies showed that stem radial growth of black spruce fluctuated with climatic factors such as summer temperature and precipitation, in which, interestingly, direction and/or strength of the climatic effects varied with site conditions such as slope position and active layer thickness (Wolken et al., 2016; Nicklen et al., 2021). In addition, another tree ring analysis showed that black spruce radial growth is also affected by soil hummock formation (mound rising) on permafrost, which causes tree leaning (Fujii et al., 2020). Considering these results, there is a possibility that the above and belowground growth of understory plants are also affected by these environmental factors directly, or indirectly through changes in overstory black spruce growth. Therefore, it is recommended that future studies will examine temporal (yearly) changes in fine root growth in relation to climatic factors and site conditions, in which it would be important to elucidate the contribution of each functional type (e.g., black spruce vs. understory shrub). Such studies will help us to understand belowground carbon flux in permafrost forests more comprehensively, and also help to predict effects of climate warming that may enhance permafrost thaw and cause shifts of plant communities (Finger et al., 2016).

## Conclusion

This study quantified fine root growth of black spruce and understory plants during two growing seasons (2018–2019) in a permafrost black spruce forest in Interior Alaska. This black spruce forest is located along a north-facing slope, where active layer thickness and aboveground biomass of black spruce trees decreased downslope in general. Our major finding was that the fine root growth rate of understory plants increased downslope, whereas that of black spruce tended to decrease. As a result, understory root growth accounted for more than 50% of total fine root growth at the low-slope plot (NE260). Furthermore, we found that *Sphagnum* mosses, which often dominate forest floor at sites with the shallow active layer, were likely to benefit fine root growth of understory plants as compared with feather mosses. In conclusion, this study suggests that fine root growth of understory plants such as Ericaceae shrubs is a key to better understanding belowground carbon dynamics in permafrost forests especially at sites with the shallow active layer.

## Data Availability Statement

The raw data supporting the conclusions of this article will be made available by the authors, without undue reservation.

## Author Contributions

KN, YM, and TM conducted the fieldwork. KN performed the root analyses in the laboratory and prepared the manuscript. JT, YM, TM, and YK contributed to discussing and editing it. All authors contributed to the planning of this study.

## Conflict of Interest

The authors declare that the research was conducted in the absence of any commercial or financial relationships that could be construed as a potential conflict of interest.

## Publisher’s Note

All claims expressed in this article are solely those of the authors and do not necessarily represent those of their affiliated organizations, or those of the publisher, the editors and the reviewers. Any product that may be evaluated in this article, or claim that may be made by its manufacturer, is not guaranteed or endorsed by the publisher.
