# Supplementary material for: Fine Root Growth of Black Spruce Trees and Understory Plants in a Permafrost Forest Along a North-Facing Slope in Interior Alaska
Source: Front Plant Sci. 2021 Nov 16;12:769710. doi: 10.3389/fpls.2021.769710 (PMC8635146; doi:10.3389/fpls.2021.769710)
Supplement: Supplementary file 1 [file Data_Sheet_1.pdf]

Supplementary Material

1 Supplementary Table

Supplementary Table 1. Results of one-way ANOVA for effects of plot on fine root growth parameters

Fine root parameter	Species	Substrate	*F*-value	*P*-value
FRG	Spruce	Feather	0.55	0.58
		Sphagnum	0.19	0.67
	Understory	Feather	**4.71**	**0.019**
		Sphagnum	0.16	0.69
	Total	Feather	0.21	0.81
		Sphagnum	0.23	0.64
FRG (proportion in 0–10 cm)	Spruce	Feather	**8.90**	**0.001**
		Sphagnum	0.02	0.90
	Understory	Feather*	–	–
		Sphagnum	0.02	0.88
	Total	Feather	**6.17**	**0.007**
		Sphagnum	0.02	0.90
FRG (understory proportion)		Feather	**5.80**	**0.009**
		Sphagnum	0.04	0.85
SRL	Spruce	Feather	0.19	0.83
		Sphagnum	0.28	0.61
	Understory	Feather	**5.60**	**0.026**
		Sphagnum	0.35	0.58
RTD	Spruce	Feather	3.47	0.076
		Sphagnum	0.00	0.97
	Understory	Feather	1.80	0.22
		*Sphagnum**	–	–
Diameter	Spruce	Feather	0.04	0.96
		Sphagnum	0.00	0.99
	Understory	Feather	**4.34**	**0.048**
		Sphagnum	0.96	0.37

* Non-parametric Steel-Dwass test was done for these data because data distribution did not support the assumption of normality and equal variance.

2 Supplementary Figures

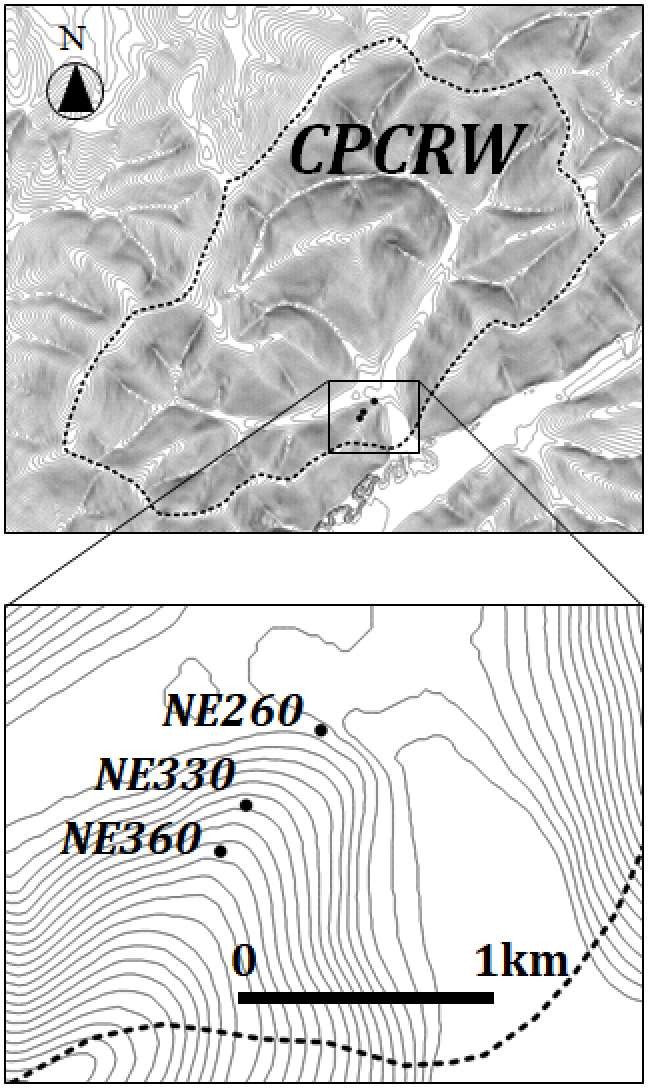

Supplementary Figure 1. Locations of our study plots in the Caribou Poker Creek Research Watershed of the University of Alaska Fairbanks. Contour lines were shown in 10 m interval. The map was created using QGIS software. GIS data were obtained from Chapin and Hollingsworth (2010).

Chapin, F.S., Hollingsworth, J., 2010. Caribou Poker Creek Research Watershed GIS Data: Digital Elevation Model (DEM). Bonanza Creek LTER, University of Alaska, Fairbanks. BNZ:435. https://doi.org/10.6073/pasta/7ebef609df2135fc249479afb5246bac. (Accessed 1 February 2021). http://www.lter.uaf.edu/data/data-detail/id/435.

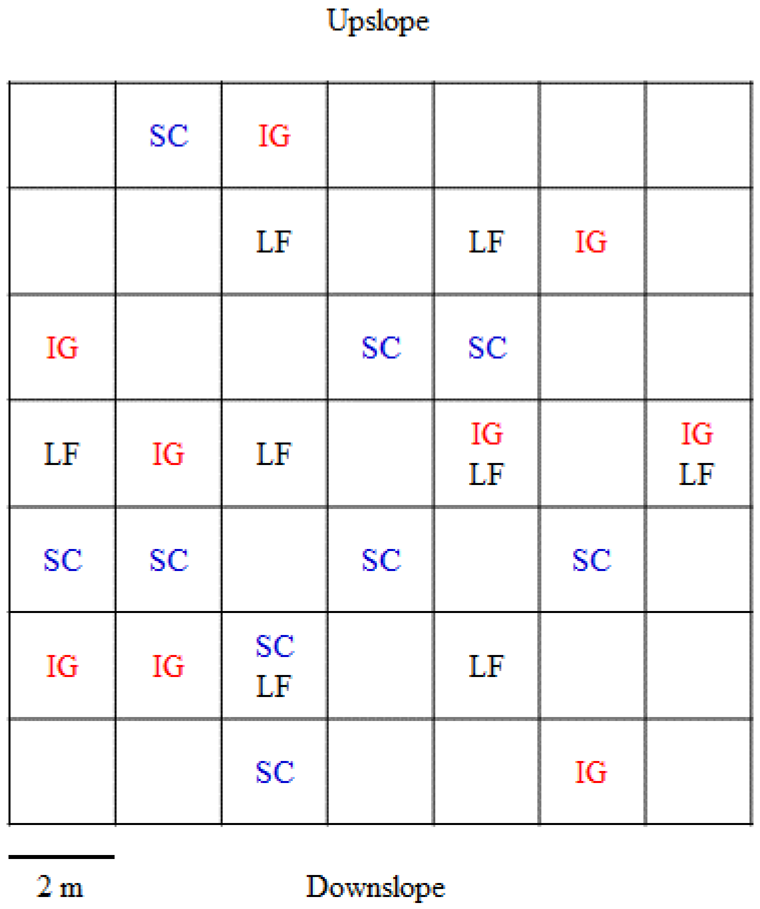

Supplementary Figure 2. Sampling locations in each study plot. The plot were divided into 49 subplots (2 m × 2 m), from which nine subplots were randomly chosen for incubation of ingrowth cores (IG) or soil coring (SC), while eight subplots were chosen for collecting litterfall (LF).

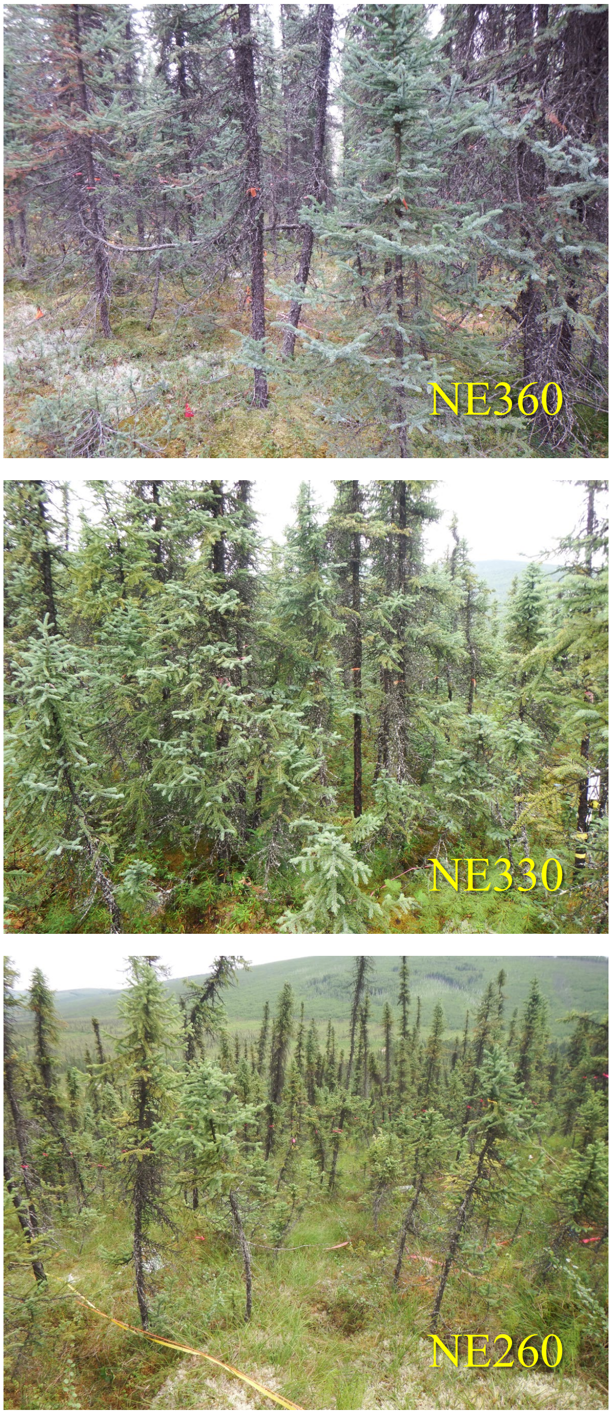

Supplementary Figure 3. Photographs of the NE360 (top), NE330 (middle) and NE260 (bottom) plots in the study site. These photographs were taken in September 2017 (NE360) and August 2018 (NE330 and NE260).

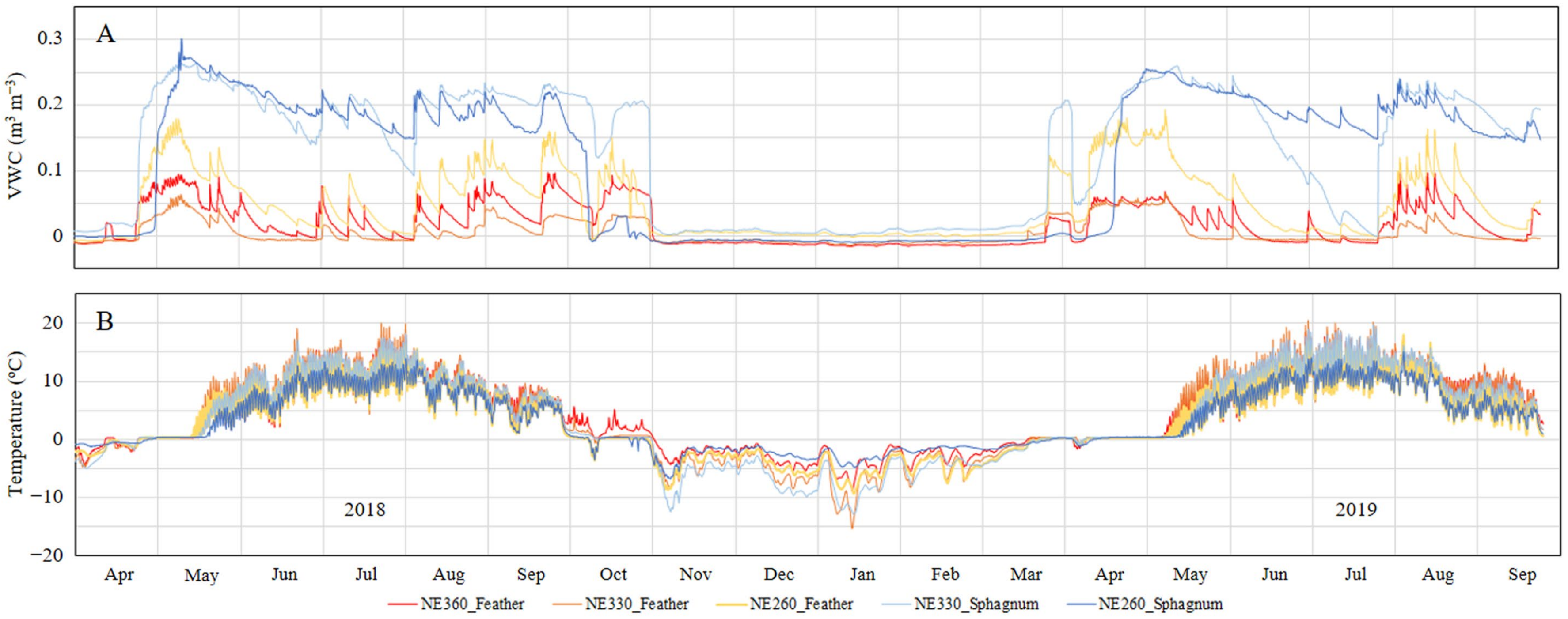

Supplementary Figure 4. Temporal changes in volumetric water contents (A) and temperature (B) at the depth of 10 cm in the organic layer. The lines represent data under feather mosses at NE360 (red), NE330 (orange) and NE260 (yellow) and under *Sphagnum* mosses at NE330 (light blue) and NE260 (blue). Data shown are values that were recorded by temperature-moisture sensors and data loggers (5TE and Em50, METER Group Inc., Pullman, WA) and calculated with calibration curves provided by the manufacturer (in default setting). Each data was obtained using one sensor (N=1).

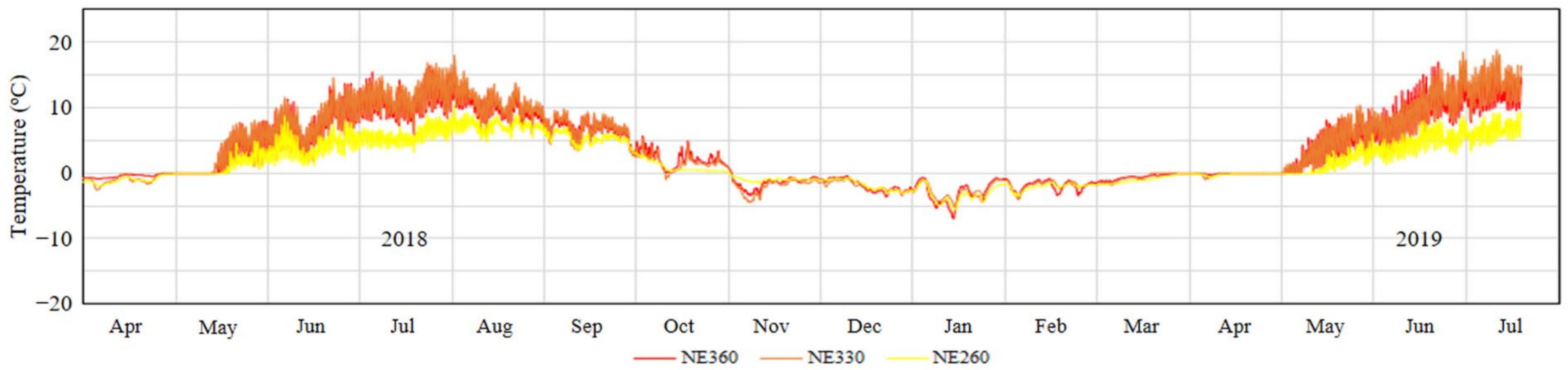

Supplementary Figure 5. Temporal changes in temperature at the bottom of organic layer (14–17 cm depth) under feather moss (and lichens). The lines represent data from adjacent stands of NE360 (red), NE330 (orange) and NE260 (yellow). Mean growing season temperature (May–September 2018) was 7.4, 7.9 and 4.7°C at NE360, NE330 and NE260, respectively. Data shown are values that were recorded by temperature loggers (TR51*i*, T and D, Matsumoto, Japan). Each data was obtained using one sensor (N=1).
